# Acute Appendicitis: Epidemiological, Clinical, Surgical, and Post-surgical Characteristics in a Honduran General Hospital

**DOI:** 10.7759/cureus.40428

**Published:** 2023-06-14

**Authors:** Reenie H Pineda Villeda, Diana L Flores Reyes, Juan F Suazo Rivera

**Affiliations:** 1 Department of General Surgery, Mario Catarino Rivas Hospital, San Pedro Sula, HND; 2 Department of Internal Medicine, Mid Yorkshire NHS Trust, Wakefield, GBR

**Keywords:** complications, disease severity, surgical intervention, epidemiology, acute appendicitis

## Abstract

Background: Acute appendicitis is a common surgical emergency worldwide, yet data specific to Central America, including Honduras, are limited. This study aimed to investigate the epidemiological, clinical, surgical, and post-surgical characteristics of acute appendicitis in a Honduran general hospital.

Methods: A descriptive, quantitative, non-experimental, cross-sectional study was conducted at the Mario Catarino Rivas Hospital in San Pedro Sula, Honduras. The study sample consisted of 100 patients admitted with acute appendicitis from January to April 2022. Data on demographic factors, surgical interventions, appendicitis phases, appendix location, and laboratory findings were collected and analyzed.

Results: The mean age of the participants was 28.5 years, with a slight male predominance (52%). Timely surgical intervention was performed in 95% of cases within the first 12 hours. The gangrenous phase was observed in 30% of patients, followed by the perforated (24%), edematous (24%), and suppurative (22%). Retrocecal appendicitis accounted for the majority of cases (66%). Moderate leucocytosis (46%) and severe leucocytosis (39%) were associated with acute appendicitis severity. A higher neutrophil percentage was indicative of complicated appendicitis. Computed tomography was underutilized, with only one patient undergoing the examination.

Conclusion: This study provides valuable insights into Honduras' epidemiological, clinical, and surgical characteristics of acute appendicitis. Early surgical intervention and laboratory findings, such as leukocyte count and neutrophil percentage, can aid in assessing disease severity. Further research is warranted to understand the unique aspects of acute appendicitis in Central America and optimize patient management. This study highlights the need for multi-centre studies and long-term follow-up to enhance our understanding of appendicitis in similar populations.

## Introduction

Acute appendicitis is the most common surgical emergency globally, with the highest incidence occurring between the second and third decades of life [1]. It involves the inflammation of the caecal appendix secondary to obstruction of its lumen, caused by fecaliths, impacted faeces, lymphoid hyperplasia, or tumours [2]. This obstruction results in oedema, vascular congestion, ischemia, and potential perforation of the appendix, which can lead to intra-abdominal abscesses or generalized peritonitis, depending on factors such as disease duration and pressure on the appendicular wall [3].

According to the Global Appendicitis Score Initiative, appendicitis incidence has significantly increased in countries undergoing industrial development, while Western hemisphere countries exhibit a decrease in cases. Some countries were excluded from the initiative due to insufficient studies to generalize statistics. Unevaluated regions include Central and South America. Specifically, Honduras was excluded from this global study due to a lack of data [4].

Although acute appendicitis is the most common indication for abdominal surgery worldwide, population data for Central and Latin America are scarce. Consequently, generalizing global data to national realities is necessary. The global annual incidence is 139.54 per 100,000 inhabitants, with a risk of 16.33% in men and 16.34% in women [3].

There is an urgent need to update the concept of epidemiological, clinical, surgical, post-surgical, and related studies associated with appendicitis in Honduras. The lack of research in the Latin American region may hinder the early identification of individuals at high risk for complications.

## Materials and methods

We designed and conducted a descriptive, quantitative, non-experimental, cross-sectional study. The study sample included all (100) patients admitted with acute appendicitis to the Mario Catarino Rivas Hospital in San Pedro Sula, Honduras, from January 1, 2022, to April 30, 2022, who met the inclusion criteria.

The objective was to identify the epidemiological and clinical behaviour of patients presenting with acute appendicitis, characterize their surgical behaviour, and analyze complementary laboratory studies.

Inclusion criteria were as follows: men and women aged 18 and over, treated for acute appendicitis, and admitted to the Mario Catarino Rivas Hospital from January 1, 2022, to April 30, 2022, with complete medical records and signed consent forms.

We collected data after obtaining approval from the local university hospital ethics committee with approval number 294 and processed it using SPSS.

## Results

Our study included 100 patients with a mean age of 28.5 years. The majority of participants were in the 20- to 25-year age range (35%, n=35), followed by young adults (28%, n=28), intermediate adults (13%, n=13), and adolescents and older adults (10% each, n=10). Elderly adults constituted the minority (4%, n=4). Males represented 52% (n=52) of the sample, and females 48%.

Regarding surgical intervention, 95% (n=95) of patients underwent timely surgical intervention within the first 12 hours. In the 13- to 24-hour range, considered lower risk of perforation, 4% (n=4) of patients received surgical treatment. One patient (1%) underwent surgery between 25 and 72 hours after admission.

In terms of perforation risk, 46% (n=46) of patients were at higher risk, presenting between 25 and 144 hours. Lower-risk patients (41%, n=41) presented between 13 and 24 hours. Twelve percent (n=12) presented at an opportune time (6-12 hours), and 1% (n=1) presented early (2-5 hours). In terms of length of hospitalization, 47% (n=47) stayed for one day post-op, while 50% (n=46) stayed from two to five days.

**Figure 1:**
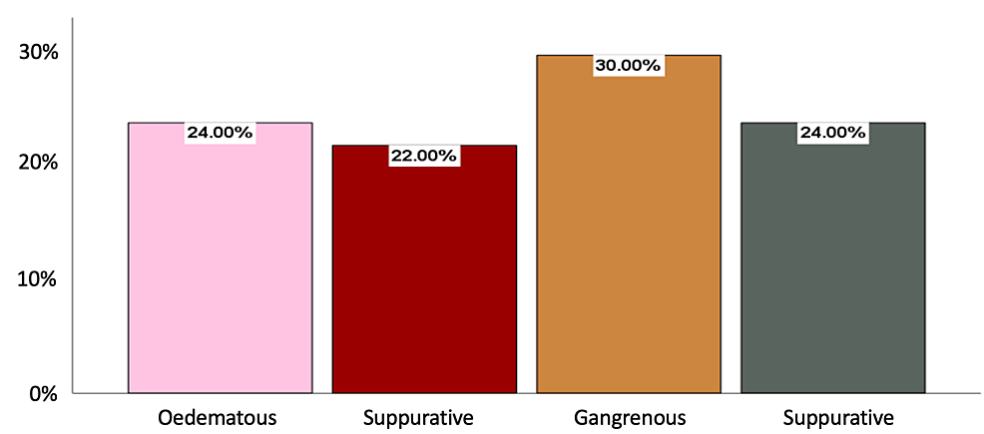
Frequency of acute appendicitis phase (n=100) in Honduran General Hospital

The most frequent comorbidities on initial presentation were being overweight (14% [n=14]), obesity (10% [n=10]), T2 diabetes mellitus (6% [n=6]), and hypertension (3% [n=3]). Regarding the acute appendicitis phase, 30% (n=30) were in the gangrenous phase, 24% (n=24) in the perforated phase, 24% (n=24) in the oedematous phase, and 22% (n=22) in the suppurative phase (Figure 1).

Regarding the type of surgery, 97% (n=97) involved open surgery with a McBurney incision (60%, n=60), followed by a mid-line infra-umbilical incision (30%, n=30). The most common complication was localized peritonitis (23%, n=23). Concerning appendix location, 66% (n=66) were retro-cecal, 9% (n=9) were pelvic, 25% (n=25) were in other positions, and 1% (n=1) were para-ileal.

**Figure 2:**
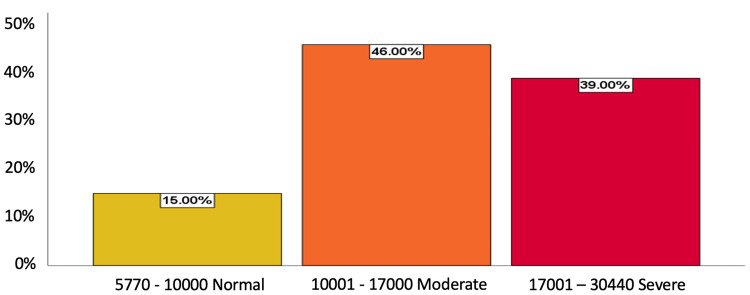
Leukocyte count of patients (n=100) with acute appendicitis in Honduran General Hospital

For leukocyte count, 46% (n=46) had a moderate leukocyte count (10,001-17,000/mm^3^), 39% (n=39) had severe leukocytosis (17,001-30,440/mm^3^), and 15% (n=15) had a normal leukocyte count (5,770-10,000/mm3). Regarding neutrophil percentage, 58% (n=58) had a percentage of 83% to 96% (indicating complicated appendicitis), 25% (n=25) had a value of 76% to 82% (uncomplicated appendicitis), and 17% (n=17) had a normal percentage (56% to 75%). In total, 43% (n=43) presented a neutrophil-lymphocyte index of 9 to 89 associated with complicated appendicitis, 42% (n=42) presented a range of 4-8 associated with uncomplicated appendicitis, and 15% (n=15) of patients showed a normal range with values of 2 and 3 (Figure 2).

In terms of urine tests, 68% (n=68) of patients did not undergo an examination. Among those examined, 20% (n=20) showed no alterations, 11% (n=11) displayed leukocytosis, and 1% (n=1) tested positive for leukocytes and nitrites. Regarding abdominal ultrasounds, 65% (n=65) did not undergo this examination. Of those examined, 17% (n=17) presented a non-compressible appendix with double wall thickness and a diameter greater than 6 mm, 5% (n=5) presented fluid in the right lower quadrant, and 3% (n=3) presented pain upon compression. As for computed tomography use, only one patient (1%) underwent this examination, which revealed peri-appendicular fat deformation and/or appendicular wall enhancement.

## Discussion

In our study, the mean age of participants was 28.5 years, aligning with the global trend of acute appendicitis being more prevalent among young adults [5]. This age distribution is also consistent with findings from the United States [6]. The age distribution in our study also mirrors that observed in other Latin American studies, such as those conducted in Mexico [7] and Colombia [8], which report the highest incidence of acute appendicitis among young adults in their twenties.

Regarding gender distribution, our study found a slightly higher proportion of male patients (52%), compared to female patients (48%). This finding is in line with previous studies reporting a slightly higher incidence of acute appendicitis in males [9]. However, it is important to note that this difference is not substantial, and acute appendicitis remains a significant health concern for both genders.

The majority of patients in our study (95%) underwent timely surgical intervention within the first 12 hours. This is a crucial factor in reducing the risk of complications, such as perforation, which can lead to more severe consequences like intra-abdominal abscesses or generalized peritonitis [9]. Our results are consistent with other studies conducted in Latin America [10] and the United States [11], which have reported a high percentage of timely surgical interventions. The timeliness of surgical interventions is important in the context of global appendicitis incidence, which helps guide the management of acute appendicitis [12].

In terms of the phases of acute appendicitis, our study found that 30% of patients were in the gangrenous phase, 24% in the perforated phase, 24% in the edematous phase, and 22% in the suppurative phase. The distribution of appendicitis phases in our study is similar to results reported in other regional studies [13]. This underscores the importance of prompt diagnosis and surgical intervention to prevent disease progression and associated complications.

Regarding appendix location, our study found that 66% of cases were retro-cecal, which aligns with previous research reporting a higher prevalence of retro-cecal appendicitis [14]. This information may be valuable for surgeons when planning the surgical approach and determining the optimal treatment strategy for each patient.

Our study also showed that 46% of patients had a moderate leukocyte count and 39% had severe leukocytosis, indicating an association between leukocyte count and the severity of acute appendicitis. This finding is consistent with other studies that have reported a correlation between leukocyte count and appendicitis severity [15]. The neutrophil percentage was also found to be associated with complicated appendicitis in our study, which is consistent with previous research [16].

## Conclusions

In conclusion, this study highlights valuable insights into acute appendicitis's clinical characteristics and management, emphasizing the importance of early surgical intervention to prevent complications. The high neutrophil percentage can serve as an indicator of complicated appendicitis. By understanding the specific characteristics of acute appendicitis in this population, healthcare professionals can better identify high-risk individuals, optimize treatment strategies, and ultimately improve patient outcomes. The study's limitations should be considered, such as its single-centre design, limited sample size, confounding factors, and lack of follow-up data. Future multi-centre studies and longer-term patient follow-up could provide additional insights into appendicitis in Honduras and similar demographic settings.
